# Risk factors for shoulder injuries in handball: systematic review

**DOI:** 10.1186/s13102-022-00588-x

**Published:** 2022-12-02

**Authors:** Stelios Hadjisavvas, Michalis A. Efstathiou, Vivian Malliou, Christoforos D. Giannaki, Manos Stefanakis

**Affiliations:** 1 Department of Life and Health Sciences, University of Nicosia, Nicosia, Cyprus; 2 Department of Physical Education and Sport Science, Democritus University of Thrace, Komotini, Greece

**Keywords:** Shoulder pain, Sports injuries, Odds ratios, Relative risk, Risk of injury

## Abstract

**Background:**

Handball is a sport with a high number of severe injuries**.** The shoulder is one of the most commonly injured joints, with an average prevalence of 17–41%.

**Objective:**

The primary aim is to identify the most significant risk factors related to shoulder injuries in handball. The secondary aim is to propose recommendations based on the available evidence concerning potential injury prevention strategies.

**Methods:**

Systematic searches of PubMed, MEDLINE, CINAHL, Proquest, SPORTDiscus, Web of Science, EMBASE, and Scopus were undertaken for peer reviewed articles published between 15 July 1995 and 15 July 2019. The same search strategy was repeated on 24 April 2022. Prospective cohort studies, written in English and published in a journal with a peer-review process aiming to investigate at least one modifiable or/and a non-modifiable potential risk factor for shoulder injuries, specifically in handball players, were included. Only papers published after 1995 were included. The methodological quality of the eligible studies was assessed using the modified version of the Downs and Black Checklist. The Best Evidence Synthesis (BES) approach was used for synthesizing and reporting the results.

**Results:**

1849 studies were identified, of which 8 were included. A total of 2536 (males = 1354, females = 1182) participants of which 2522 were handball athletes, were included. Four of the eight studies were rated as high methodological quality studies (> 85%) while the rest were rated as medium (50–85%). The risk factors for shoulder injuries in handball identified in the studies were strength imbalances (n = 6), glenohumeral range of motion (ROM) imbalances (n = 5), scapular dyskinesis (n = 5), incorrect dosage of training load (n = 2), previous injury (n = 1), sex (n = 2), player’s position, school grade, playing level (n = 1), altered shoulder joint position sense (n = 1).

**Conclusion:**

Overall, from all the risk factors evaluated, there was strong evidence that the weakness of the shoulder external rotator muscles and the female sex increase the probability of shoulder injury in handball athletes. Nevertheless, the evidence for the other risk factors was moderate due to the methodological quality and the limited number of studies.

*Protocol registration:* PROSPERO ID: CRD42020138233.

## Key points

- Several risk factors for shoulder injuries were identified specifically in handball players
- Isometric external rotation strength was the only modifiable risk factor supported by strong evidence.
- Further research is required as only 50% of the studies were of high quality
- Future studies should address the multifactorial nature of shoulder injuries in handball.

## Background

Handball is one of the most popular team sports around the world, especially in Europe. In European Handball Federation (EHF) over 900 matches are conducted per season [1, 2]. According to the EHF website there are 50 member and two associate member federations. Each federation consists of between 4 and 18 male and female teams. Each team consists of at least 16 players (registered players per match), which means that between 3328 and 14,976 male and female participants play the sport, in the first league only, around Europe. In comparison to other sports, handball is in the top five in terms of the number and significance of total injuries [3–5]. Senior male handball players match incidence range from 15 to 73.6 injuries/h match exposure compared to senior female handball players which is 13–36 injuries/h match exposure [6, 7] Matches are characterized by repeated bouts of high-intensity activity with frequent contact and collision between players. A handball player performs at least 48,000 throwing motions with a ball weight of 425–475 g and an average throwing speed of 130 km/h over the entire season [8]. Biomechanical studies have reported that the force exerted on the throwing shoulder during the throwing motion can be up to 1.5 times the body weight of the individual [9–11]. Because of this overload, the shoulder is one of the most commonly injured joints, with up to 30% of these injuries being acute, 38% being overuse, and 45% being persistent in nature [12–14]. In addition, other studies on handball players reported shoulder pain prevalence of between 19 and 36% at the beginning of the season, and an average weekly prevalence of about 28% during the season [15, 16]. Additionally, it is estimated that 48% of handball players who report persistent shoulder problems are unable to participate in games or training due to severe pain [17].

Due to these high rates of shoulder injuries, prospective cohort and cross-sectional studies investigated several potential risk factors for shoulder injuries in handball. These studies provide important information that may guide the development of prevention programs. However, no systematic reviews have investigated the risk factors for shoulder injuries and the methodological quality of the associated studies specifically in handball players. Previous systematic reviews related to this topic [18–26] investigated only one risk factor for shoulder injuries, such as scapular dyskinesis [18, 25] training volume [24], glenohumeral internal and external rotation deficit [20, 21, 23] in various overhead athletes. Therefore, there is a need for a systematic review dealing with the whole spectrum of potential risk factors for shoulder injuries in handball.

The main aims of this study were to identify the most significant risk factors related to shoulder injuries in handball, recognize which of these are modifiable, assess the risk of bias in the relevant studies, and evaluate the evidence of the identified factors. The secondary aim is to propose recommendations based on the available evidence concerning potential injury prevention strategies.

## Μethods

This systematic review was conducted according to the Preferred Reporting Items for Systematic Reviews and Meta-Analyses (PRISMA) Guidelines [27]. The PRISMA statement includes a 27-item checklist aimed at improving reporting of systematic reviews and meta-analyses [27]. The study protocol was prospectively registered (PROSPERO ID: CRD42020138233).

### Literature search

A systematic review of peer-reviewed literature written in English, evaluating risk factors for shoulder injuries in handball players was performed using PRISMA guidelines and checklists. The following electronic international databases were searched: Pubmed, Medline complete, Cinahl, Proquest, Sport Discus, Web of Science, EMBASE, and Scopus. All databases were searched from 15 July 1995 to 15 July 2019 for peer-review papers, using the keywords (shoulder injur* or shoulder pain*) and (risk factor* or predispose* factor or contributing factor* or predict* or determin* or cause* or etiology) and (handball* or overhead athletes) using the Boolean ‘AND/OR’ operators. Both MESH terms and free text words were included in this search. The words overhead and handball were tested with the # option to test if alternative ways of writing these words existed and the results were the same. The results were inserted in a reference manager (RefWorks, ProQuest LLC) for removal of duplicates, screening, and selection. Abstract screening and selection were done independently by the two reviewers. The reference list of the included studies were reviewed for other studies not identified in the original search. The studies ought to be in English and be published after 1995 and before the date the search commenced. The year 1995 was arbitrarily chosen as the lower time limit for the literature search in order to include papers with contemporary methodology, which abide with modern publication standards. So we limited our search to around 25 years which is a time interval long enough to include all relevant studies with more or less modern methodology. The search was repeated on 24 April 2022 just before the final preparation of the publication in case further studies were published during the period of data collection and analysis. Grey literature was searched from OpenGrey.eu, as well as the following clinical trial registries: EU Clinical trials Register, Clinical Trials.gov, WHO International Clinical Trials Registry Platform, and the Australian New Zealand Clinical Trials registry.

### Eligibility criteria

The study selection criteria specifically referring to PICO are shown in Table 1. Prospective cohort studies that were published from July 1995 until July 2019, written in English language and published in a journal with a peer-review process that aimed to investigate at least one modifiable or/and a non-modifiable potential risk factor for shoulder injuries specifically in handball players were considered for inclusion in the study. The participants or population of the included studies should have the following characteristics: (1) Handball players, (2) No medical restrictions to participating in handball training or competition, (3) More than 8 h of training per week, (4) Absence of any musculoskeletal problem in the upper extremity during the period of initial testing, (5) No history of fracture or surgery to either upper extremity. Moreover, handball players were regarded as all the participants that trained at least 8 h/week (training time and competitive game time per week during the competitive period). All levels of competition were accepted (1st division, 2nd division, etc.). Comparison with other overhead athletes, comparison with other throwers, other athletes in general, and comparison with non-athletes or the general population were acceptable. The main outcome measures were the odds ratios (OR) or relative risk ratios (RR) or prevalence risk ratios (PR) or hazard rate ratios (HR) to correlate the risk factors with shoulder injuries in handball players.

**Table 1:** Inclusion and exclusion criteria

Inclusion criteria	Exclusion criteria
Studies	No prospective cohort study Previous shoulder surgery Previous glenohumeral dislocation Glenoid labrum tear Rotator cuff tear Fracture in the shoulder region in the last 6 months
Prospective Cohort Studies Peer-reviewed, English language 1995—today Assess at least one modifiable or/and a non-modifiable risk factor for shoulder injuries in handball players	
Population	
Handball players of all sexes and ages No medical restrictions Absence of any musculoskeletal problem in the upper extremity before being enrolled in the study	
Exposure	
Handball exposure at least 8 h/week (training time and competitive game time per week during the competitive period) All levels of competition are accepted (1^st^ division, 2^nd^ division, etc.)	
Comparator	
Other overhead athletes or throwers Other overhead athletes in general Non-athletes General population	
Outcome measures	
Odds Ratios (OR) Relative Risk Ratios (RR) Prevalence Risk Ratios (PR) Hazard Rare Ratios (HR)	

### Quality assessment

The same two authors assessed the methodological quality of each included study, using a modified version of the Downs and Black Checklist, independently [28]. The Downs and Black checklist is recommended in The Cochrane Handbook for assessing non-randomized trials. It is also the most widely used and well-validated tool for assessing both randomized and non-randomized trials [29]. The checklist has been shown to have good intra-rater and inter-rater reliability [28, 30]. The original version of the checklist consists of 27 questions, but for this review, several questions were excluded as not relevant. Questions 4, 8, 9, 13, 14, 15, 17, 19, 23, 24, 26 and 27 refer specifically to intervention studies and were deemed irrelevant. The rest of the items were retained, because they are specific to the aims, methods, data, and results of the studies (questions 1, 2, 3, 5, 6, 7, and 10), concern the external validity (questions 11 and 12) or the internal validity and bias of the studies (questions 16, 18, 20, 21, 22, and 25). The same modification has been used in the past in a systematic review looking at factors associated with heel pain [31]. Higher quality assessment scores signify that a higher percentage of the criteria were satisfied. Any disagreements regarding the methodological quality of the studies between the two authors (SH and MS) were first discussed and a concensus was seeked. If no agreement was reached, a third reviewer (CG) made the final decision. No cases of unresolved disagreement existed but nevertheless the third reviewer confirmed the final decision of all the included studies.

### Data extraction and analyses

The titles and/or abstracts of studies retrieved using a comprehensive search strategy and those from supplementary sources were screened independently by two reviewers (SH and MS) based on the inclusion and exclusion criteria outlined above. The full text of the potentially eligible studies was retrieved and independently assessed for eligibility by the two reviewers. Any case of disagreement was resolved through discussion.

A standardized form was used to extract suitable data from the included studies to assess of the risk of bias and synthesize the evidence. The form was custom-made in excel in advance of data extraction and the fields were the heading of Table 5. Extracted information included study setting; study population and participant demographics, baseline characteristics; details of the exposure and control conditions; study methodology; recruitment and study completion rates; outcomes and times of measurement; any other information for the assessment of the risk of bias. Two reviewers (SH and MS) extracted the data independently, and discrepancies were identified and resolved through discussion.

### Strategy for data synthesis

Τhe heterogeneity (different factors, different cut-off values, different assessment tools, etc.) of the studies and the data (lack of 2 × 2 table in most studies, ORs calculated via logistic regression models with different inputs) did not allow for a meta-analysis. A graphical representation of the results as presented in the study is shown wherever possible but without any attempt to calculate summary statistics. Instead, a qualitative assessment using the best evidence synthesis (BES) was used to formulate conclusions (Table 2). This method has been used in the past by other systematic reviews [18, 19] and consists of five levels of scientific evidence [32–34]. Consistency was defined a priori as over 75% of studies agreeing on the same direction of results.

**Table 2:** Classification of evidence-based on best-evidence synthesis approach

1. Strong evidence: evidence provided by two or more high-quality studies and by generally consistent findings across these studies (≥ 75% of the studies reported consistent findings)
2. Moderate evidence: evidence provided by one high-quality study and/or multiple studies of acceptable quality and by generally consistent findings (≥ 75% of the studies reported consistent findings)
3. Limited evidence: evidence provided by one study of acceptable quality and/or one or more studies of borderline quality
4. Conflicting evidence: inconsistent findings in multiple studies (< 75% of the studies reported consistent findings)
5. No evidence: no admissible studies were found

## Results

### Search results and selection

**Fig. 1:**
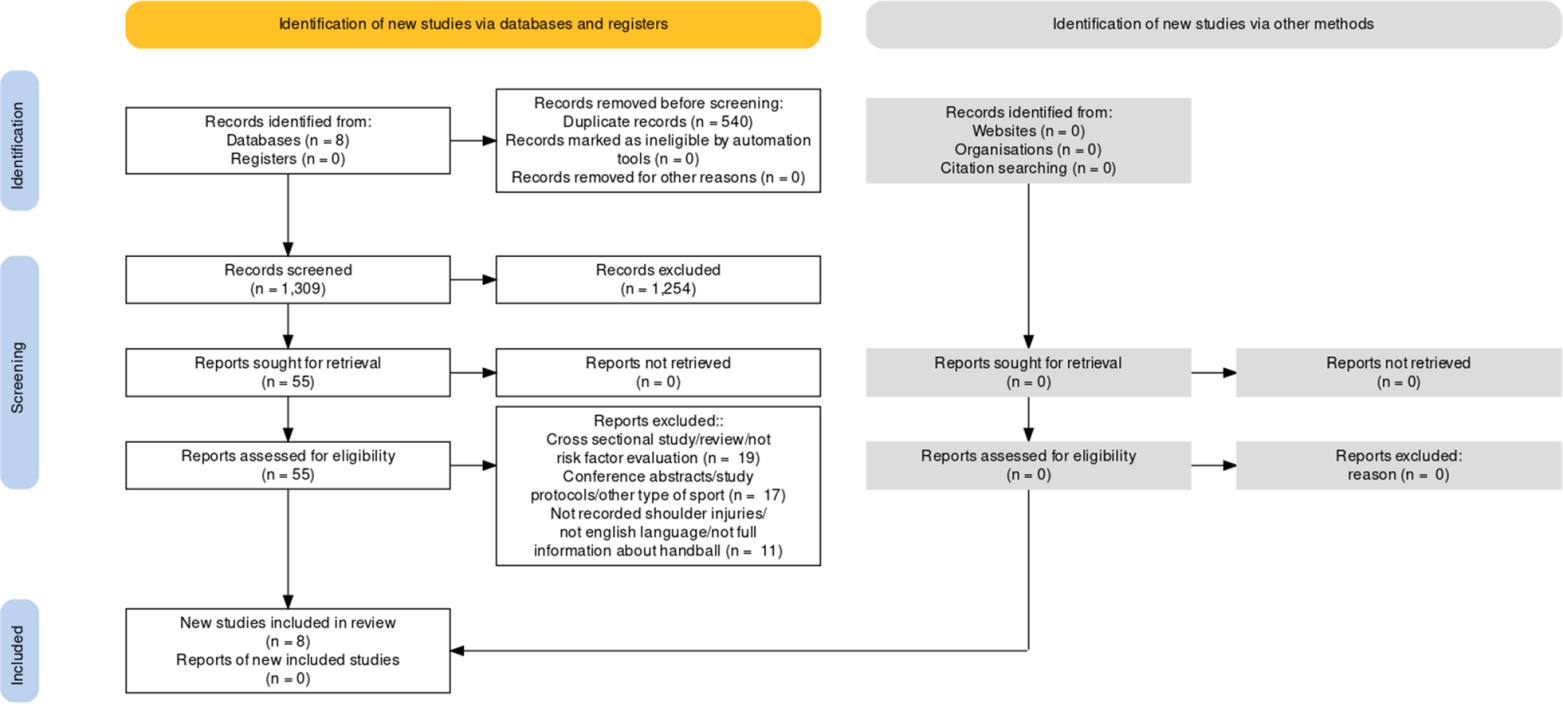
Flow chart of the included studies

The initial search identified 1849 studies (Fig. 1). Removal of duplicates eliminated another 540 studies while the title and abstract screening removed another 1254 studies. 55 full-text studies were reviewed, of which eight met the inclusion criteria and were included.

### Methodological quality

#### Modified version of the Downs and Black checklist

Quality assessment scores for the included studies ranged between 12 and 14 (Table 6). Percentage scores ranged between 75 and 88% (Mean = 82%, SD = 6.4). Four of the eight studies [16, 17, 35, 36] were rated as high methodological quality studies (> 85%) while the rest of the studies [37–40] were rated as moderate methodological quality studies (50–85%). Three studies [16, 37, 40] did not clearly report the hypothesis/aim/objective of the study, four studies did not report the distribution of principal confounders [35, 37–39], three studies [17, 37, 39] did not report actual probability values in the main outcome measures. Three studies [36, 38, 40] did not report adequate adjustment for confounding in the analyses and one study [39] did not use accurate main outcome measures.

### Study and participant characteristics

Table 3 summarizes the characteristics of the included studies. A total of 2536 (males = 1354, females = 1182) participants of which 2522 were classified as handball players based upon the methods of this study. One study (n = 16) had the smallest sample of handball players while the sample of the other studies ranged from 138 to 679 handball players [mean (SD) = 317 (201), median (IQR) = 334 (187)] (Table 3). Two studies investigated only male [16] or female athletes [37]. The age of the participants ranged between 14 and 24 years old. Four studies included adolescent athletes [17, 36, 38, 40]. Two studies did not report dropouts during the follow-up period [36, 37]. The handball experience of the athletes in four studies ranged between 8.2 and 14 years while four studies did not report the experience of their subjects [35, 37–39]. Two studies [35, 36] did not report the player’s position.

**Table 3:** Demographic characteristics of the studies

Study ID	Sample (N)	Females	Males	Handball players
Edouard et al. [37]	30	30	0	16
Clarsen et al. [16]	206	0	206	206
Achenbach et al. [36]	138	68	70	138
Giroto et al. [35]	339	183	156	339
Asker et al. [17]	471	256	215	471
Moller et al. [38]	679	304	375	679
Andersson et al. [39]	329	161	168	329
Asker et al. [40]	344	180	164	344
***Mean (SD)***	***317 (201)***	***148 (107)***	***169 (110)***	***315 (204)***

The studies used different definitions for shoulder injuries except for two studies [16, 39]. Moller et al. [38] assessed any new shoulder injury in the dominant arm, «defined as any handball-related shoulder problem irrespective of the need for time loss or medical attention». In the study by Edouard et al. [37] the shoulder injury was registered when the player consulted the National team physician and «was unable to take full part in handball activity or match play at least one day beyond the day of injury». Αll acute, traumatic, and overuse injuries were analyzed. One study [39] assessed only overuse shoulder problems in the dominant arm, defined as «any pain, ache, stiffness, instability, looseness, or other symptoms related to the shoulder, affecting the player’s participation, training volume and performance, as well as the presence of pain over the previous 7 days». Importantly they excluded acute injuries. The same group in a previous study [16] calculated the prevalence of shoulder pain in both arms using the same operational definition and included in the analysis only those with moderate to severe restrictions in training, performance, or participation. In the study by Asker et al. [17] shoulder problems were categorized into two types, any shoulder problems, and substantial shoulder problems. If a problem was reported in any of the four questions in the modified Swedish OSTRC overuse injury questionnaire was categorized as any shoulder problem. For substantial shoulder problems, the authors used the same definition as Clarsen et al. [16], namely «problems leading to moderate or severe reductions in training volume, or sports performance, or complete inability to participate in sport». Giroto et al. [35] used different definitions for new injuries, previous injuries, overuse injuries, contact injuries, injuries without contact, injury severity and recurrent injuries. Importantly in their analysis, they compared three groups: non-injured athletes (reference group), athletes reporting a new traumatic injury, and athletes reporting a new overuse injury. In the study by Achenbach et al. [36], the overuse shoulder injuries and re-injuries were used in the analysis and were defined as «injuries with no identifiable traumatic event and injuries sustained at the same body site within two months after the first injury respectively». Asker et al. [40] defined shoulder injury as reporting a score of 40 or more (OSTRC questionnaire) from the dominant shoulder at some point during the season. Furthermore, the authors had to modify the OSTRC overuse injury questionnaire in order to collect information about shoulder problems during the past two months and the past season (the original questionnaire collects data the past week only).

The risk factors for shoulder injuries in handball identified in the included studies were: strength imbalances [16, 36–40], glenohumeral range of motion imbalances [16, 36, 38–40], scapular dyskinesis [16, 36, 38–40], incorrect dosage of training load [35, 38], previous injury [35], sex [17, 35], player’s position, school grade, playing level [17] and altered shoulder joint position sense [40].

Four of the eight studies used the Oslo Sports Trauma Research Center (OSRTC) overuse injury questionnaire [16, 17, 39, 40] to record shoulder injuries in handball. One study [36] used an online questionnaire that addressed any overuse injuries during a handball training session or match and the Western Ontario Shoulder Index (WOSI). One study [35] used a weekly injury questionnaire that collected the date/situation of injury, and the injury in match/training. The Sports Injury Surveillance (SPEx) system was used in the study of Moller et al. [38]. Finally, in the study of Edouard et al. [37], all new shoulder injuries in youth players were recorded by the national team physician when a player consulted him/her for pain or injury or by medical interview by the physician every month. In the assessment of risk factors, four studies [16, 35, 36, 39] used the odds ratios (O.R), two studies [38, 40] the hazard rate ratios (H.R), one study [37] the relative risk ratio (R.R) and one study [17] the prevalence rate ratios (P.R).

### Synthesis of results

#### Muscle strength imbalances

**Fig. 2:**
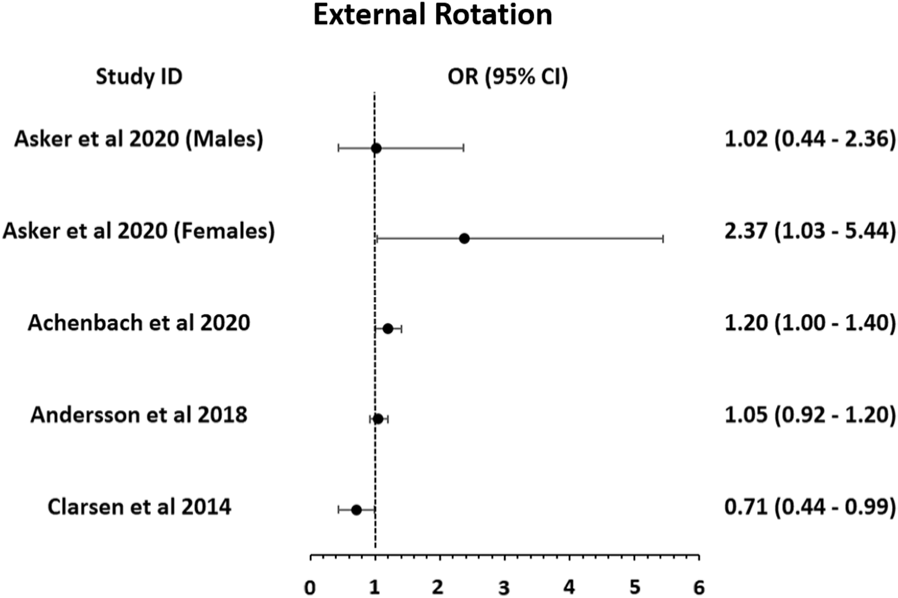
Graphical representation of the results for isometric ER strength as presented in the studies. (*Note*: Asker et al. calculated Hazard ratios instead of ORs)

Muscle strength imbalances as a risk factor for shoulder injuries in handball players were examined in two high-quality studies [16, 36] and four moderate-quality studies [37–40]. One moderate quality study (limited evidence) [37] found that female handball players with low ratios of concentric ER to concentric IR strength at 240°/s, and high ratios of eccentric IR to concentric ER strength at 60°/s have a 2.5 times higher risk of overuse and acute shoulder injury (p < 0.05). Three out of four high-quality studies (strong evidence) agree there is an association between decreased ER isometric strength and overuse shoulder injuries in males (p from 0.034 to 0.046) [16, 36] and females (p = 0.034) [36, 40]. Only one study [39] found no significant association (p = 0.45) between external rotation strength and overuse shoulder injury (Fig. 2). There was limited evidence (one moderate quality study) that reduced external rotation strength exacerbates the association between increased (increase between 20 and 60% per week) handball training load and overuse or acute shoulder injury among elite youth handball players (p = 0.04) [38].

#### Glenohumeral ROM imbalances

**Fig. 3:**
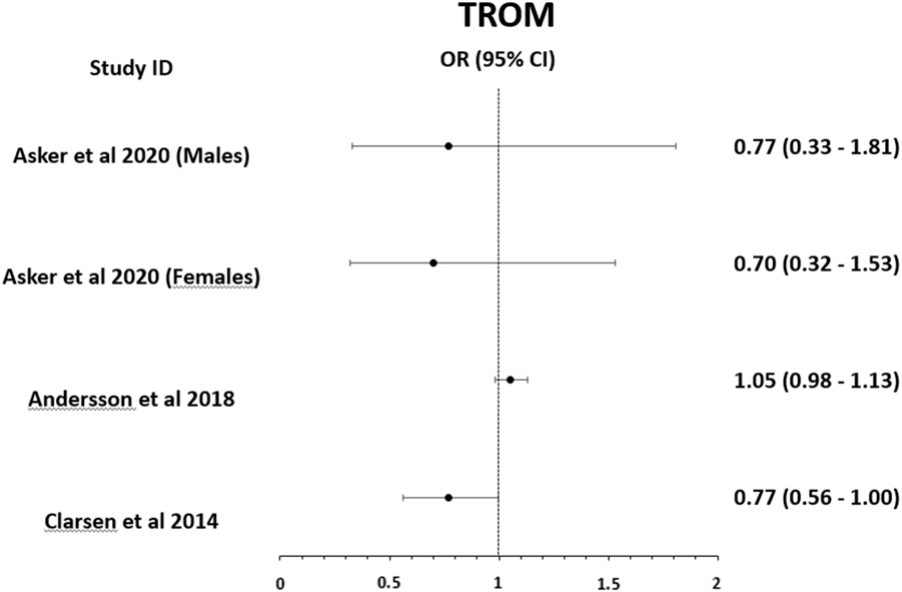
Graphical representation of the results for the TROM as presented in the studies. (*Note*: Asker et al. calculated Hazard ratios instead of ORs)

The association between various glenohumeral ROM imbalances and shoulder injuries was examined by two high-quality studies [16, 36] and three medium-quality studies [38–40]. There was moderate evidence that glenohumeral ROM imbalances and total rotational range of motion (TROM) are not significantly (p values between 0.15 and 0.92) associated with shoulder injuries [38–40]. There was limited evidence that absolute TROM values rather than TROM differences (Fig. 3) are significantly (p = 0.046) associated with overuse shoulder problems in elite male players only [16]. There was limited evidence that greater internal rotation ROM is significantly (p = 0.046) associated with overuse shoulder injury [39]. There was moderate evidence that an increased external rotation motion of more than 7.5° (p = 0.025) and a glenohumeral internal rotation deficit (GIRD) of more than 7.5° (p = 0.014) are risk factors for an overuse shoulder injury in youth female players [36].

#### Scapular dyskinesis

**Fig. 4:**
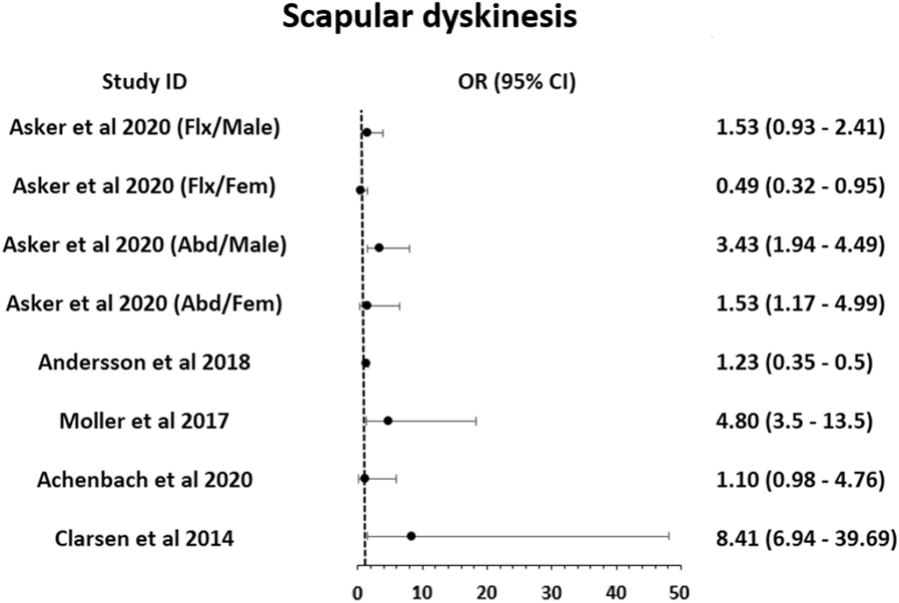
Graphical representation of the results for scapular dyskinesis as presented in the studies. Studies (*Note*: Asker et al. and Moller et al. calculated Hazard ratios instead of ORs)

Scapular dyskinesis was examined in two high-quality studies [16, 36] and three moderate-quality studies [38–40]. Overall 60% (3/5) of the studies point toward the direction of a significant association (p = 0.02) between shoulder injuries and scapular dyskinesis (Fig. 4). Three studies found that obvious scapular dyskinesis is a risk factor for overuse [16, 38, 40] and acute shoulder injuries [38]. Two studies found no significant association (p = 0.80) between scapular dyskinesis and overuse shoulder injury [36, 39]. Overall there was conflicting evidence regarding the role of scapular dyskinesis in shoulder injuries in handball. There was limited evidence that scapular dyskinesis exacerbated the association between increased handball load and overuse or acute shoulder injury among elite youth handball players (p = 0.02) [38].

#### Shoulder joint position sense

The association between shoulder joint position sense and shoulder injury was examined in one medium-quality study [40]. There was limited evidence that there is no association between joint position sense and overuse shoulder injuries in both male and female players.

#### Training and match load

Excessive handball training and match loads were examined in one high-quality study [35] and one medium-quality study [38]. There was moderate evidence that an increase in handball load with an additional match per week is associated with overuse shoulder injury [35]. Moreover, there was limited evidence that an increase in handball load by > 60% is associated with a greater shoulder injury rate (p = 0.05) and this association is stronger in players with reduced external rotation strength (p = 0.01). Smaller increases in handball load between 20 and 60% are relevant only among players with reduced external rotational strength (p = 0.04) or scapular dyskinesis (p = 0.02) [38].

#### History of injury

The association between the history of injury with current shoulder injuries in handball players was examined in one high-quality study [35]. There was moderate evidence that the previous injury is associated with a higher risk of an overuse shoulder injury.

#### Sex

The association between sex and shoulder injuries was examined in two high-quality studies [13, 15]. There was strong evidence that the female sex has higher odds of traumatic shoulder injuries and a higher prevalence of shoulder injuries.

#### Player’s position, school grade, playing level

The player's position, school grade, and playing level were examined in one high-quality study [17]. There was moderate evidence that the prevalence of shoulder injuries is significantly higher in backcourt players, but no differences were found for school grade or playing level. Table 4 summarizes the most important findings of this review, while Table 5 contains the full details of the studies included (Table 6).

**Table 4:** Summary of the main results

Independent Factors	Category	Injury type	Evidence of support	Grade of evidence	Details
Muscle imbalances	Isokinetic Ratios	Acute and overuse	One moderate quality study	Limited evidence	Low conER/conIR strength at 240°/s and high eccIR/conER strength at 60°/s had a 2.5 times higher risk
	Isometric ER strength	Overuse	Four high-quality studies	Strong evidence	Decreased ER strength increases the risk
ROM imbalances	ER-IR ROM imbalance	Any shoulder injury	Two moderate-quality studies	Moderate evidence	No association with shoulder injuries
	TROM	Any shoulder injury and overuse injuries	Three moderate-quality studies	Moderate evidence	No association with shoulder injuries
	Greater IR	Overuse	One moderate quality study	Limited evidence	16% increased risk per five degrees change
	ER > 7.5° and GIRD > 7.5°	Overuse	One high-quality study	Moderate evidence	Higher risk in youth female players
Scapula Dyskinesis	Obvious scapula dyskinesis	Acute and overuse	Two high-quality and three moderate-quality studies	Conflicting evidence	Three out of five studies found a positive association
Joint position sense	Joint position error	Any shoulder injury	One moderate quality study	Limited evidence	No association with shoulder injuries
Workload	One additional match/week	Overuse	One high-quality study	Moderate evidence	On average 31% increased risk for injury
	> 60% increase in training load	Overuse	One moderate quality study	Limited evidence	Increased risk of injury compared to those with < 20% increase in training load
History of injury		Overuse	One high-quality study	Moderate evidence	
Sex	Females	Acute	Two high-quality studies	Strong evidence	Higher prevalence of shoulder injuries and higher odds of traumatic injuries
Player’s position	Backcourt	Any shoulder injury	One high-quality study	Moderate evidence	Higher prevalence compared to 6 m players
School grade	1st, 2nd, or 3rd year student	Any shoulder injury	One high-quality study	Moderate evidence	No association with shoulder injuries
Playing level	Regional vs national	Any shoulder injury	One high-quality study	Moderate evidence	No association with shoulder injuries

Interaction of Factors	Injury type	Evidence of support	Grade of evidence	Details
ER strength and increased workload	Acute and overuse	One moderate quality study	Limited evidence	Decreased ER strength increases the rate of shoulder injuries when load increases 20–60%/week or > 60%/week
Obvious scapular dyskinesis and increased workload	Acute and overuse	Two moderate quality study	Limited evidence	Obvious scapula dyskinesis increases the rate of shoulder injuries when load increases 20–60%/week

**Table 5:** Characteristics of the included studies

Authors	Study type	Follow up duration	Recruited	Population characteristics	Dropouts (reason)	Risk factors (Method of evaluation)	Method of shoulder injury reporting	Association of risk factors with shoulder injuries
Edouard et al. [37]	Prospective Cohort Study	Season 2009–2010	16 F elite handball players and 14 healthy F non-athletes (Control group)	Age (18 years, SD 1) Height (174 ± 6 cm) Weight (70 ± 9 kg) BMI (23 ± 2 kg.m) Right-handed (n = 11) Workload: Mean 10 h—per week and 28 matches per year Goalkeepers (n = 2), Wingers (n = 5) Backcourts (n = 5) Centres (n = 3) Pivot (n = 1)	NR	Rotator muscle strength imbalances (Isokinetic dynamometer). Muscular imbalance if ≥ 2 of the following criteria existed: dominant side deficit of ≥ 10% in concentric (at 60, 120, or 240°/s) and/or in eccentric (at 60°/s); ER/IR ratio below 0.63 at 60°/s; ER/IR ratio below 0.64 at 120°/s; ER/IR ratio below 0.69 at 240°/s; ERecc/IRcon ratio below 0.67; and IRecc/ERcon ratio above 1.61 at 240°/s	All new shoulder injuries were recorded by the national team physician and/or by monthly physician's medical interview. Injury was defined as the player being unable to participate fully in training or match at least one day past the day of injury	A significant association was found between muscle imbalance (ERcon/IRcon at 240°/s and IRecc/ERcon at 60°/s) and shoulder injury (RR 2.57, 95% CI: 1.60–3.54; *P* < 0.05)
Clarsen et al. [16]	Prospective Cohort Study	Season 2011–2012 (September 2011 to May 2012)	206 M elite handball players	Age (24 years, SD 4) Height (189 cm) Weight (89 kg) Handball experience (14 years and 4 years in elite series) Right-handed (73%) Back players (42%) Wing players (23%) Line players (15%), Goalkeepers (14%) Combination of positions (6%)	N = 42 (inadequate response to questionnaire excluded from the analysis)	1) TROM (digital inclinometer) 2) Isometric ER, IR, and abduction strength (digital handheld dynamometer) 3) Scapular control (during 5 repetitive flexion and abduction movements with 5 kg). Rating: normal control, slight dyskinesis, obvious dyskinesis	OSTRC Overuse Injury Questionnaire (email every 2 weeks)	Obvious dyskinesis (OR 8.41, 95% CI 1.47 to 48.1, p < 0.05), total rotational ROM (OR 0.77 per 5° change, 95% CI 0.56 to 0.995, p < 0.05) and ER strength (OR 0.71 per 10 Nm change, 95% CI 0.44 to 0.99, p < 0.05) were significant risk factors
Achenbach et al. [36]	Prospective Cohort Study	Season 2017–2018 (7 months)	138 (70 M and 68 F) elite youth handball players	Age (14.1 years, SD 0.8) Height (175.2 ± 8.2 cm) Weight (64.0 ± 9.6 kg) BMI (21.1 ± 2.0) Team handball experience (8.2 ± 2.2 years)	NR	1) GIRD, TROM, ER gain (manual goniometer) 2) ER strength, IR isometric and eccentric strength, ERecc/IRecc, and ER isometric/IR isometric (hand-held dynamometer) 3) Scapular movement (overhead movements while holding a 2-kg dumbbell. Rating as present or absent and absent, moderate or severe scapular dyskinesis 4) Maximum throwing velocity (stationary radar gun)	Data were collected at 5 time points using the same questionnaire: after baseline screening, before beginning of the season, after the preseason, during mid-season, and at the end of the season	Decreased absolute, isometric ER strength and normalised isometric and eccentric ER strength, as well as the ER:IR strength ratio were significant risk factors for overuse injury. ER gain of > 7.5° and GIRD of > 7.5° were also significant risk factors for an overuse injury in girls. Scapular dyskinesis and maximum throwing velocity were not different among players with or without overuse injury
Giroto et al. [35]	Prospective Cohort Study	One season	339 elite handball athletes (156 M and 183 F)	Age (23.4 years, SD 4.6) Weight (76.7 kg) Height (1.77 m) BMI (24.2 kg/m^2^) Right-handed (84.9%) Left-handed (15.1%) Previous injuries last 6 months (46.6%)	N = 27 (the reason is not reported)	1) Sex 2) Age 3) BMI 4) Previous injuries 5) Exposure (hrs/week) to strength training 6) Number of matches/week	Weekly exposure questionnaire was filled out by one person (in each team) responsible to provide data	History of injury (OR: 2.42, 95% CI: 1.51–3.89), and an extra match per week (OR: 1.31, 95% CI: 1.05–1.62) were risk factors for overuse injuries. Female athletes (OR: 1.56, 95% CI: 1.08–2.25), and an extra hour of training per week (OR: 1.09, 95% CI: 1.02–1.15) were risk factors for traumatic injuries. Age and BMI were not significant
Asker et al. [17]	Prospective Cohort Study	One season (2014–2015 or 2015–2016 season	471 elite adolescent handball players (215 M and 256 F)	Age (16.4 years, SD 0.85) Height (176.8 cm) Weight (74.15 kg) BMI (23.8) Year of playing handball (9.1 years) Goalkeepers (n = 72) Wing players (n = 90) Line players (n = 63) Backcourt players (n = 241) National level (n = 119) Regional level (n = 352)	N = 26 (Quit handball, long-standing injury not in the shoulder, unable to continue, unknown reason)	1)Sex 2)Player’s position 3)School Grade 4)Playing level	The Swedish version of the OSTRC overuse injury questionnaire (every week)	Higher prevalence in female (PR 1.46, 95% 1.04–2.06) and backcourt players (PR 1.58, 95% CI 1.08–2.32). School grade (PR 1.21 95% CI 0.88–1.67) or playing level (PR 1.09 95% CI 0.76–1.56) were not significant risk factors
Moller et al. [38]	Prospective Cohort Study	One season (2013- 2014)	679 elite youth handball players (304 F and 375 M)	Age (16 years, SD 2) Back players (n = 303) Wing players (n = 168) Line players (n = 102) Goalkeepers (n = 97) History of shoulder injury (n = 43)	N = 8 (excluded from analysis as they did not report any handball participation)	1) Handball load changes: (a) < 20% increase or decrease, (b) 20% to 60% increase or decrease (c) > 60% increase 2) Scapular control (normal or obvious scapular dyskinesia) 3) Rotational isometric strength (ER:IR ratio dominant arm at 0 and 30 degrees of rotation) and abduction strength (hand-held dynamometer) 4) Glenohumeral ROM side to side differences in TROM, IR, and ER	Injury and participation information were collected weekly by SMS and a telephone interview, and a physical examination by medical personnel within 1–2 weeks following an injury	Significantly higher injury rate with a > 60% increase in load (HR 1.91; 95% CI 1.00 to 3.70). This was more pronounced when reduced ER strength co-existed (HR 4.2; 95% CI 1.4 to 12.8). If handball load increased between 20 and 60% rate of injury was higher only when players had reduced external rotational strength (HR 4.0; 95% CI 1.1 to 15.2) or scapular dyskinesis (HR 4.8; 95% CI 1.3 to 18.3)
Andersson et al. [39]	Prospective Cohort Study	One season (2014–2015)	329 elite handball players (168 M and 161 F)	Age (14 years, SD 5) Right-handed (78%) Backs (41%) Wings (25%) Line players (15%) Goalkeepers (13%) Multiple positions (6%)	N = 39 (withdrawn, retired from handball, pregnant, acute injury rest of season)	1) Glenohumeral IR and ER ROM (digital inclinometer) 2) Isometric IR and ER rotation strength (handheld dynamometer) 3) Scapular dyskinesis (5 reps of flexion and abduction with extra weight). Rating: normal control, slight dyskinesis or obvious dyskinesis	OSTRC Overuse Injury Questionnaire (six time points in the whole season)	No significant associations between total rotational ROM, external rotation strength, or obvious scapular dyskinesis and overuse shoulder injury. Greater internal rotation was associated with overuse injuries (OR 1.16 per 5° change, 95% CI 1.00 to 1.34)
Asker et al. [40]	Prospective Cohort Study	One or two seasons (2014–2015 and/or 2015–2016)	344 students handball players (180 F and 164 M)	Age (16.55 years, SD 0.85) Height (176.6 cm) Weight (74.7 kg) Handball experience (9.35 years) Goalkeepers (17%) Wing players (20.5%) Line players (15.5%) Back players (47%) 1^st^ grade (47.5%) 2^nd^ grade (35%) 3^rd^ grade (17.5%) History of shoulder pain (31.5%) Regional playing level (76%) National playing level (24%)	N = 127 (Absent during baseline test days, unable to perform shoulder tests, reported an OSTRC score of ≥ 40 the past 2 months, no response to any of the weekly questionnaires)	1) Shoulder strength (isometric ER, IR, abduction) and eccentric ER (handheld dynamometer) 2) Shoulder ROM (digital inclinometer) 3) Shoulder joint position sense (supine position with target angle at 75% of the maximum ER) 4) Scapular dyskinesis (Each player performed two repetitions of maximum shoulder abduction and maximum shoulder flexion in random order with weights (1kgr for female and 2kgr for male). Videos were reviewed by one tester for presence or absence of scapular dyskinesia separately for abduction and flexion)	A modified version of OSTRC overuse injury questionnaire (every week)	Isometric external rotation strength in female players (HR = 2.37, 95% CI 1.03- 5.44) but not in male palyers (HR = 1.02, 95% CI 0.44–2.36) was found significant. Same results for isometric internal rotation strength (Female HR = 2.44, 95% CI 1.06–5.61 and male HR = 0.74, 95% CI 0.31–1.75). There was no association between ROM and shoulder injuries for both sexes. Scapular dyskinesia during abduction was significant only in male players (female HR = 1.53, 95% CI 0.36–6.52, male HR = 3.43, 95% CI 1.49–7.92. Scapular dyskinesia in flexion and joint position sense were not significant factor for shoulder injuries in both sexes

F, females; M, males; n, number of participants; NR, not reported; ER, external rotation; IR, internal rotation; ROM, range of motion; °/s, degrees per second; Ecc, eccentric; Con, concentric; TROM, total range of motion; GIRD, glenohumeral internal rotation deficit; BMI, body mass index; OSTRC, Oslo Sports Trauma Research Center; SPEx, Sports Injury Surveillance; OR, odds ratios; PR, prevalence ratios; RR, risk ratios; HR, hazard ratios P, probability value; CI, confidence intervals

**Table 6:** Quality assessment scores for included studies

Questions																	
Study	1	2	3	5	6	7	10	11	12	16	18	20	21	22	25	Total	%
Edouard et al. [37]	0	1	1	0	1	1	0	1	1	1	1	1	1	1	1	12	75
Clarsen et al. [16]	0	1	1	1	1	1	1	1	1	1	1	1	1	1	1	14	88
Achenbach et al. [36]	1	1	1	1	1	1	1	1	1	1	1	1	1	1	0	14	88
Giroto et al. [35]	1	1	1	0	1	1	1	1	1	1	1	1	1	1	1	14	88
Asker et al. [17]	1	1	1	1	1	1	0	1	1	1	1	1	1	1	1	14	88
Moller et al. [38]	1	1	1	0	1	1	1	1	1	1	1	1	1	?	0	12	75
Andersson et al. [39]	1	1	1	0	1	1	1	1	1	1	1	0	1	1	1	13	81
Asker et al. [40]	0	1	1	1	1	1	0	1	1	1	1	1	1	1	0	12	75

1: criterion satisfied, 0: criterion not satisfied, ?: unable to decide

## Discussion

The purpose of this systematic review was to investigate risk factors for shoulder injuries in handball players. In comparison to other overhead sports (e.g. baseball, volleyball, softball, tennis), there is a small number of studies looking at handball. Overall, 8 prospective cohort studies were analyzed in this review [16, 17, 35–40]. Several potential risk factors were investigated in these studies. However, strong evidence was found only for the weakness of the external rotator cuff muscles and the female sex that they increase the probability of shoulder injury in handball athletes.

### Μuscle strength imbalances and shoulder injuries

Three out of four high methodological quality studies agree that the decreased isometric strength of the shoulder external rotator muscles is significantly associated with overuse shoulder injuries in handball athletes [16, 36, 39, 40]. The result seems to be independent of the method used to assess it and the variation in the sample among the positive studies. For example, there are differences in the mean ages of the participants. The average age of handball players in the studies was 24 years [16], 14 years [36], and 17 years [40]. In addition, there are differences in the initial position of assessing muscle strength. In the study of Clarsen et al. [16] the participants were placed in a supine position, with the shoulder in a neutral position and with the elbow flexed in 90^ο^. Instead, in the study of Asker et al. [40] the seated position was preferred with the shoulder abducted in 30^ο^. On the other hand, Achenbach et al. [36] did not mention the initial position of the participants. Furthermore, in the study by Andersson et al. [39], although ER strength was not confirmed as a significant risk factor, male athletes were stronger than their female counterparts and showed a lower percentage of shoulder injuries and substantial shoulder injuries despite having a significantly higher exposure to handball training. Taken together these results suggest that isometric external rotation strength is an important variable to monitor both pre-season and during the season.

Studies have found that the strength of the rotator cuff muscles of the shoulder may be affected by the initial position of the shoulder due to the length-tension relationship of the muscles and the tension of the ligaments and the joint capsule [41–44]. Forthomme et al. [42] reported that the ability of the rotator cuff muscle to produce power was greater at the 90^ο^ of shoulder abduction compared to the 45^ο^ of abduction. In the study of Lin et al. [44] when the ratios of internal and external rotator muscle strength were evaluated at different abduction angles, rotational power was greater at the 70^ο^ of shoulder abduction [44]. Several studies choose to evaluate the rotator cuff muscle strength in shoulder positions below 90^ο^, however, ball throwing most often takes place around 90^ο^ of abduction. Therefore, it is suggested that the evaluation of rotator cuff strength is more sport specific at 90^0^ of abduction.

Biomechanically, the shoulder external rotator muscles play an important role in stabilizing the shoulder during the cocking phase of ball throwing. If there is a weakness in the shoulder external rotator muscles, the humeral head is believed to slide upwards especially during ball throwing, due to the action of the deltoid muscle. This reduces the subacromial space, potentially leading to compression of the supraspinatus tendon [45, 46]. However, this traditional view of subacromial compression causing the pathophysiology of shoulder problems is not supported by evidence [47]. Specifically, humeral migration seems to be the result of rotator cuff deficiency not the cause of it [48], bilateral full-thickness tears often cause unilateral problems [49], contact between the rotator cuff and the coracoacromial arch is common in asymptomatic subjects [50, 51], there is no correlation between acromiohumeral distance and pain and function in patients with rotator cuff disease [52] and the most common side of partial tears is the articular not the bursal [53, 54] aspect of the tendon.

A more plausible explanation for shoulder pain in overhead athletes is overload. During ball throwing, the deceleration of the internal rotation of the shoulder after the ball leaves the hand depends largely on the shoulder's external rotator muscles [5]. Strength deficits of the external rotators will result in rotator cuff overload and reduced ability to decelerate the movement [55]. There are also reports of a decrease in the strength of the external rotators in the dominant shoulder with a simultaneous increase in the strength of the internal rotator and the adductor muscles [56]. This results in muscle imbalance between the shoulder's internal and external rotator muscles and the possibility of shoulder injury [57]. In the study of Edouard et al. [37], it was found that the lower ratios of muscle strength between concentric external rotation and concentric internal rotation and the higher ratios between eccentric internal rotation and concentric external rotation were associated with a 2.5 times higher risk of overuse injury and acute shoulder injuries in women. A similar result was found in the study of Achenbach et al. [36], where the lower ratios of muscle strength between shoulder internal and external rotator muscles were associated with a higher risk of shoulder overuse injury in male handball players.

Up to 15% differences in internal rotation strength between the dominant and non-dominant sides as found in the study by Edouard et al. [37] are considered a normal adaptation to sport [43, 58]. The strength difference between internal and external rotators in the dominant arm is possibly the result of resistance training emphasizing the internal rotator muscles (pectoralis major, latissimus dorsi, teres major) due to the need to produce high internal rotation power during throwing. In addition, repeated throws of the ball can lead to an adaptive increase in the strength of the shoulder's internal rotator muscles. This increase of the internal rotation strength helps the athlete achieve faster ball speed, resulting in higher ball throwing efficiency [59, 60] but can increase the risk of injury when not sufficiently balanced with the decelerating ability of the external rotators. Studies by Andrade et al. [61] in handball players and Wang and Cochrane [62] in volleyball players suggest that functional ratios (ERecc/IRcon), should be greater than 1. This reference value can be useful in developing fitness and rehabilitation programs, especially because it was associated with fewer shoulder injuries at least in volleyball players [62]. Although Edouard et al. [37] did not confirm this suggestion in handball players, since they found reduced functional ratios in handball players and controls, it might still be valid due to the methodological differences between the two studies such as different isokinetic velocities, different sex of the participants and different functional ratios in the non-dominant arm.

### Glenohumeral ROM imbalances and shoulder injuries

Handball players like other throwing athletes have a greater ΕR ROM to the dominant shoulder compared to the non-dominant shoulder [13, 15, 16, 63–66]. This greater range of motion during the cocking and acceleration phases is potentially related to faster ball throwing [67]. However, increased external rotation of the shoulder is believed to cause retroversion of the humeral head due to the rotational loads, particularly when these occur in an immature skeleton [68, 69]. Consequently, most overhead athletes present with increased external rotation and reduced internal rotation ROM in the dominant arm (Glenohumeral Internal Rotation Deficit—GIRD) [64]. This deficit in the internal rotation often results in a reduction in overall shoulder rotation (total range of motion-TROM) and this is believed to increase the risk of shoulder injury in overhead athletes [65]. An alternative explanation for the reduced TROM is considered the posterior capsular and muscular tightness of the glenohumeral joint [68, 70, 71].

The glenohumeral ROM imbalances as a possible risk factor for shoulder injuries in handball players were investigated in 5 studies [16, 36, 38–40]. In general, the role of ROM imbalances and TROM in shoulder injuries was not supported by the studies included in this review. Clarsen et al. [16] suggested that absolute TROM values rather than TROM differences were significantly associated with overuse shoulder problems in elite male players but a subsequent study by the same group, with the same methodology, in a larger sample of male and female players, failed to confirm this finding and suggested that only greater internal rotation ROM was associated with overuse shoulder injuries [39]. Only the study by Achenbach et al. [36] found a significant correlation between increased external rotation and GIRD in the dominant shoulder with overuse shoulder injuries in female athletes. An explanation for the discrepancy with other overhead athletes [72] might be that ball throwing in handball is performed with various techniques (e.g. overarm and sidearm throw) [73] compared to other throwing sports, and the shoulders of handball players are often exposed to contact and blockage while in an elevated position [16].

### Scapular dyskinesis and shoulder injuries

Scapular dyskinesis is common in people with shoulder pain and various shoulder pathologies such as impingement syndrome, rotator cuff tears, glenoid labrum tears, and instability [74–77]. It is also common finding in other overhead athletes, such as baseball players, swimmers, and tennis players [78–80]. However, it is frequent finding in symptom-free athletes [76, 81–83], and an association between scapular dyskinesis and shoulder symptoms among overhead athletes has been been esteblished [16, 76, 83]. No clear evidence was found in the present study regarding the association between scapular dyskinesis and shoulder injuries in handball due to the conflicting results, the limited number of studies, and the methodological differences between studies. In contrast, Hickey et al. [18] in the systematic review that they conducted, concluded that overhead athletes who present scapular dyskinesis have a 43% higher risk of causing shoulder pain compared to athletes who do not have scapular dyskinesis. However, only one study with handball players was included in this systematic review. In another, more recent systematic review Ηοgan et al. [25] found that scapular dyskinesis was not a significant risk factor for a shoulder injury in throwing athletes from different sports (e.g. baseball, rugby).

In the present study, 3 out of 5 studies show that scapular dyskinesis is a significant risk factor for a shoulder injury in handball players [16, 38, 40]. Perhaps the conflicting results arise due to the lack of consensus in assessing scapular dyskinesis. In the study by Clarsen et al. [16] external weight (5 kg) was used for the assessment of scapular dyskinesis and participants performed 5 repetitions of flexion and abduction while holding a weight. In contrast, in the study by Asker et al. [40] a smaller external weight (2 kg for men and 1 kg for women) was used for 2 repetitions of flexion and 2 repetition of abduction while holding a weight. Other studies did not provide sufficient information on how to assess scapular dyskinesis. Another source of variation is the categorization of dyskinesis with some studies using a binary classification (present or not) [40] and others using grades of severity (normal / mild dyskinesia / obvious dyskinesia) [16]. It has been suggested that the evaluation of two options (normal or abnormal) is more reliable [84]. Furthermore, some studies used video while others did not [36] to assess scapular dyskinesia. Until these methodological issues are resolved, the relationship between scapular dyskinesis and shoulder injuries will probably produce conflicting results.

### Training load and shoulder injuries

Evidence on the training load and its relationship to injuries in throwing athletes is in its early stages and most of the research is currently on baseball, cricket, football, rugby, and volleyball [85–89]. In the present review, only two included studies evaluated the effect of training load on shoulder injuries in handball players [35, 38]. In the study by Giroto et al. [35], it is apparent that the addition of an official match per week is significantly associated with overuse shoulder injuries. In the study of Moller et al. [38] players who increased their weekly handball training load by 60% or more, were twice more likely to suffer an overuse injury compared to players that increased their training load by 20%.

Another significant finding of the study by Moller et al. [38] was the interaction of training load with other potential risk factors such as obvious scapular dyskinesis and reduced external rotation strength. These moderators seem to exacerbate the association between increased training load and overuse injuries. These findings emphasize the multifactorial nature of athletic injuries and the importance of complex, nonlinear interaction between different factors, which requires a more dynamic system approach in injury prevention research [90, 91].

### Shoulder joint position sense and shoulder injuries

The association of reduced shoulder proprioception with shoulder injuries in handball athletes was investigated in only one moderate methodological quality study [40]. However, no significant association was found between shoulder proprioception and shoulder injuries. One limitation of this study is that only one subcategory of proprioception was assessed (joint position sense). As it is well known, proprioception is divided into other subcategories, such as the sense of movement (kinesthesia), the sense of force, the sense of change in velocity, and the sense of vibration [92, 93]. Further research is required to determine the effect of reduced proprioception in shoulder injuries in handball. This is especially important after an injury that is generally believed to disrupt proprioception [94, 95].

### History of previous injury and shoulder injuries

A history of a previous injury is arguably one of the most important non-modifiable risk factors associated with various sports injuries. Examples of sports injuries that have been significantly correlated with a history of a previous injury are hamstring strains [96–98], ankle sprains [99], shoulder dislocations [100], and groin injuries [101]. In the present review, only one high-quality study investigated the history of a previous injury as a possible risk factor for a future shoulder injury in handball athletes [35]. Aligned with the results in other injuries, professional handball players are 2.5 times more likely to sustain a new shoulder injury when they have a history of a previous injury. The study by Moller et al. [102] also reported that a history of 2 or more previous injuries is a significant risk factor for future injury in 16-year-old handball players. However, all previous injuries (locations and types) were included in the analysis, and this makes it difficult to isolate the results for the shoulder. Some studies show that a previous injury, such as an ankle sprain, increased the risk of developing a new and more serious injury in the same area [103, 104]. Inadequate recovery from previous injuries could explain the reason that a previous injury is a significant risk factor for causing future injuries.

### Sex and shoulder injuries

Another non-modifiable risk factor that is commonly associated with sports injuries is sex. Women athletes have been found to have higher rates of concussion [105] and ACL injuries [106, 107] than men. In the present study, two high methodological quality studies [17, 35] agree that adolescent and mature female handball players have a greater risk of shoulder injuries than male players. Michalsik and Aagard [108] found that female handball athletes are subjected to greater relative workload compared to men. On the contrary, the same study found that male players engage in more physical and strenuous confrontations and perfrom more high intensity work during the game. Perhaps the higher relative workload in females results in fatigue, which increases the risk of injury, while the higher intensity work in male players results in improved fitness, which is protective from injuries. In addition, kinematic differences in ball throwing have been found between men and women. Men perform ball throwing with a greater rotational speed of the trunk and with a greater range of motion of horizontal shoulder abduction during the cocking phase while women demonstrate greater external rotation of the shoulder during the cocking phase [109]. In addition, male handball players have a higher ball throwing speed on the wrist and hand compared to women [110]. Perhaps these and known anatomical differences [111] result in better leverage, significant higher ball coverage index, better utilization of the whole kinetic chain and lower relative loads on a stronger skeleton which collectively account for the differences in the risk of injury between the sexes.

### Player’s position and shoulder injuries

In one high methodological quality study [17], a significant correlation between backcourt handball players and shoulder injuries was found. Other studies suggest the same finding concerning other injuries such as ACL injuries [106]. This is perhaps because backcourt players have the highest incidence of injuries and the highest frequency of acute, non-contact, lower limb injuries compared to other player positions as Wedderkopp et al. [103] showed. One possible explanation is that backs show a higher number of shots and passes compared to other positions [5]. These activities involve a significant deceleration and acceleration action into abduction and external rotation, which potentially increases the risk of injury [36]. In addition, backcourt players engage in more aggressive contacts compared to players in other positions [5].

### Limitations

The present study has some limitations. First, only published studies that were written in English were used. Second, the small number of included studies, the differences in the risk factors assessed, and the considerable variability in methods and sample characteristics made it difficult to combine them in a meta-analysis. The heterogeneity of the studies also provided considerable difficulty in the interpretation of the results and the derivation of solid suggestions for injury prevention. Finally, the possibility of not including some relevant papers of considerable interest cannot be excluded, as our literature search was limited up until 1995.

#### Limitations of the included studies

The studies included in this review suffer from several limitations. The operational definition of shoulder injury was not universal. In addition, several studies looked at only overuse and not at all shoulder injuries. Even the studies that looked at all shoulder injuries did not provide adequate separation between overuse and acute injuries as risk factors might be different for these types of injuries. There was very limited or no information on the specific diagnosis of the injuries included in each study. There was no universally agreed method to record new shoulder injuries between studies and this creates a challenge to compare the results. Some studies suffer from a considerable loss of data (e.g. on a weekly basis) and therefore the number of injuries might be lower than the true one. Most studies evaluated individual risk factors but as already mentioned and confirmed by the study by Moller et al. [38] the cause of athletic injury is usually multifactorial. Therefore, the results should be interpreted with caution.

### Directions for future research

More studies are necessary that specifically investigate the risk factors for a shoulder injury in handball players. In addition, more studies should be conducted on younger handball players to understand the potentially modifiable risk factors to prevent shoulder injuries and extent the athletic career. Future studies should also include both female and male handball players so that differences in risk between sexes can be determined. There should be a consensus regarding technical (e.g. position of measurements) and methodological issues (e.g. definition of injury, assessment of scapular dyskinesis, method of injury surveillance) to produce solid results and avoid confusion.

### Clinical suggestions and practical applications

Based on the results of this systematic review the following are suggested for the prevention of shoulder injuries in handball players:

1. Athletes should be evaluated with reliable measuring tools (e.g. isokinetic dynamometer or handheld dynamometer) during the pre-season period to identify any strength deficits or imbalances and intervention should be applied to correct them.
2. The average weekly increase in training load should be monitored to avoid overuse. Important moderators such as scapular dyskinesia or weakness of the external rotators should be corrected as they make the athlete vulnerable to injury even with lower increases in training load.
3. It is recommended for handball athletes who have suffered a serious shoulder injury to perform a complete and correct recovery to enhance the mechanical strength of injured tissues at pre-injury levels. In addition, injured athletes need to have reliable and measurable assessment tools (eg isokinetic dynamometer) before returning to their sport after injury.
4. Based on the studies by Giroto et al. [35] and Moller et al. [38] we conclude that increases in workload (Hrs/week) below 20% are unlikely to increase the risk of shoulder injury.
5. All of the recommendations are probably more important for backcourt and female players

## Conclusions

Several risk factors for shoulder injuries in Handball players were identified. Strong evidence was found for one modifiable (ER strength) and one non-modifiable risk factor (female sex). Moderate evidence was found for glenohumeral ROM imbalances, incorrect dosage of training load, previous injury, player’s position, school grade and playing level. Training load in particular seems to be related to shoulder injuries both independently and by interacting with other factors such as ER strength and scapular dyskinesis.

## Data Availability

Detailed search results are available on request (stefanakis.m@unic.ac.cy).

## Abbreviations

PRISMA: Preferred reporting items for systematic reviews and meta-analyses
PICO: Patient/population, intervention, comparison, and outcomes
OR: Odds ratios
RR: Relative risk ratios
HR: Hazard rate ratios
PR: Prevalence risk ratios
BES: Best evidence synthesis
OSRTC: Oslo Sports Trauma Research Center
SPEx: Sports injury surveillance
ER: External rotation
IR: Internal rotation
ROM: Range of movement
TROM: Total range of movement
GIRD: Glenohumeral internal rotation deficit
F: Females
M: Males
n: Number of participants
NR: Not reported
Ecc: Eccentric
Con: Concentric
p-value: Probability value
CI: Confidence intervals
